# Supplementary material for: Ca2+ transients on the T cell surface trigger rapid integrin activation in a timescale of seconds
Source: Nat Commun. 2024 Jul 20;15:6131. doi: 10.1038/s41467-024-50464-0 (PMC11271479; doi:10.1038/s41467-024-50464-0)
Supplement: Supplementary file 1 — Supplementary Information [file 41467_2024_50464_MOESM1_ESM.pdf]

Supplementary information

Ca^{2+} transients on T cell surface trigger integrin rapid activation on a second timescale

Li et al

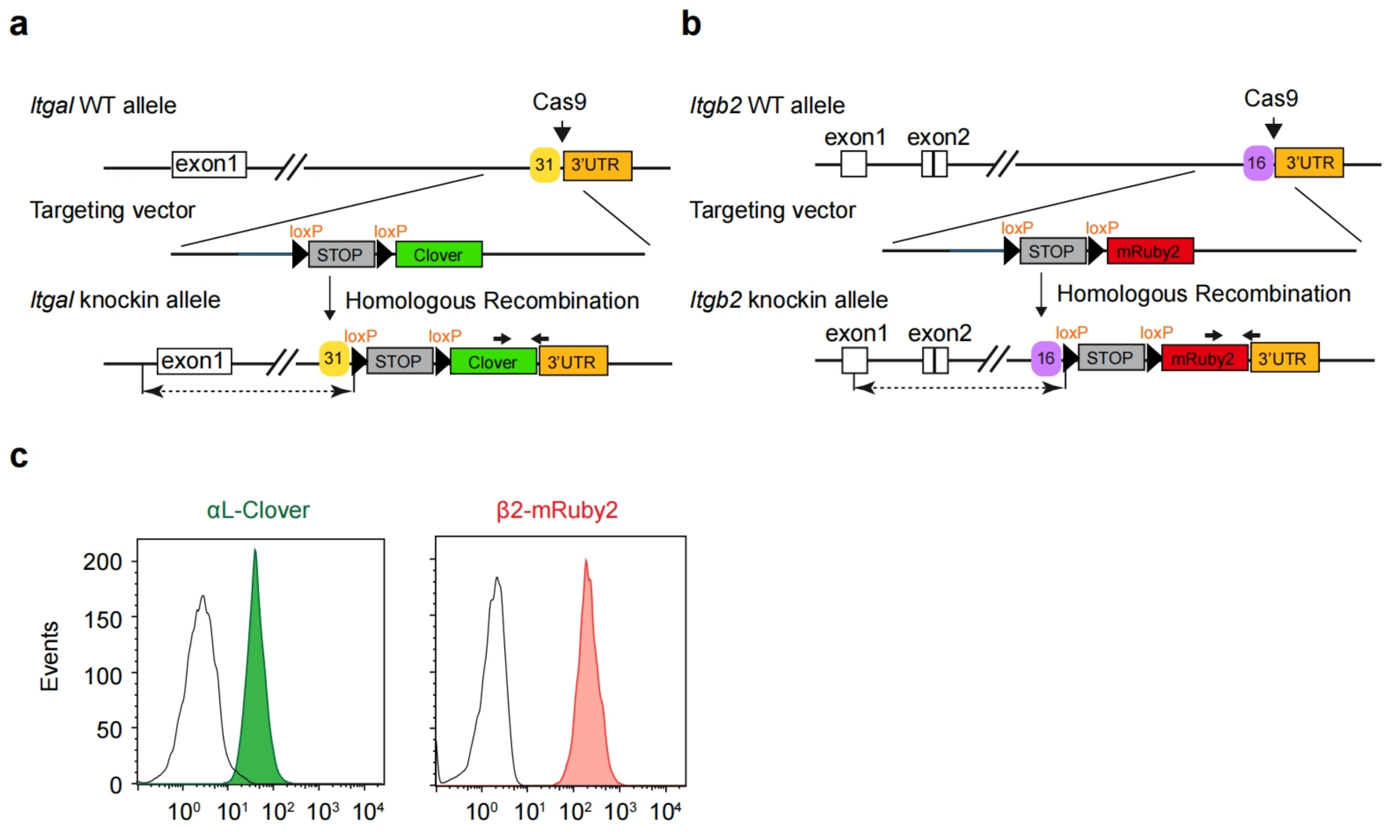

Supplementary Fig. 1: Generation of *Itgal-LSL-Clover* and *Itgb2-LSL-mRuby2* mice.

a-b, The strategies for generation of *Itgal-LSL-Clover* (**a**) and *Itgb2-LSL-mRuby2* (**b**) knock-in alleles by homologous recombination using CRISPR/Cas9.

c, The expression of αL_{Clover} and $\beta 2_{mRuby2}$ in the splenic T cells from *Itgal-LSL-Clover*;*Itgb2-LSL-mRuby2*;*CD4-Cre* mice was detected by flow cytometry analysis. T cells from WT mice were used as control (open histogram).

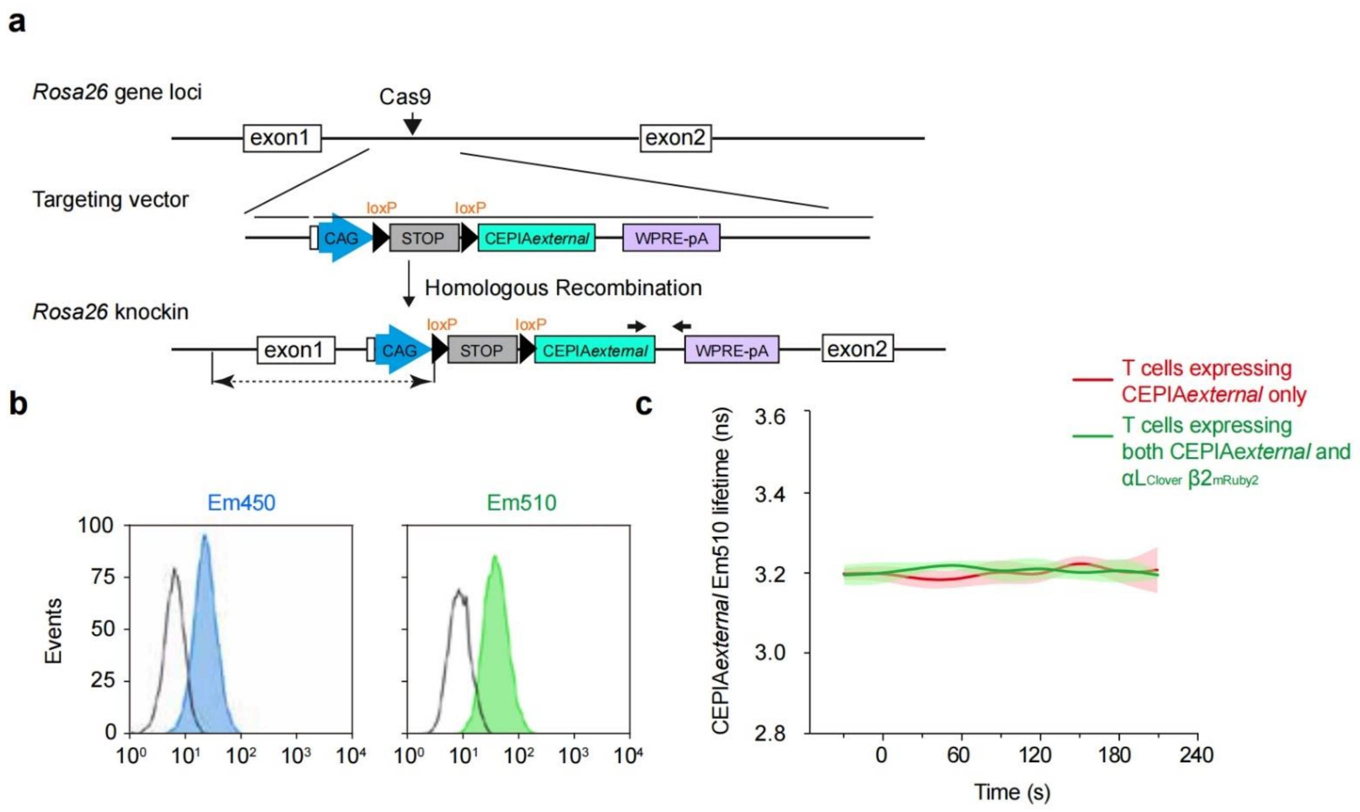

Supplementary Fig. 2: Generation of *R26-LSL-CEPIAexternal*;*Itgal-LSL-Clover*;*Itgb2-LSL-mRuby2*;*CD4-Cre* mice.

a, Generation of *R26-LSL-CEPIAexternal* mice. The strategy for generation of *R26-LSL-CEPIAexternal* knock-in allele by homologous recombination using CRISPR/Cas9.

b, *R26-LSL-CEPIAexternal* mice were crossed with *Itgal-LSL-Clover*;*Itgb2-LSL-mRuby2*;*CD4-Cre* mice to obtain *R26-LSL-CEPIAexternal*;*Itgal-LSL-Clover*;*Itgb2-LSL-mRuby2*;*CD4-Cre* mice carrying T cells expressing CEPIA*external* and $\alpha L_{Clover}\beta 2_{mRuby2}$ simultaneously. The expression of CEPIA*external* in splenic T cells from *R26-LSL-CEPIAexternal*;*Itgal-LSL-Clover*;*Itgb2-LSL-mRuby2*;*CD4-Cre* mice was detected by flow cytometry analysis. T cells from WT mice were used as control (open histogram).

c, Time-lapse FLIM measurement of CEPIA*external* Em510 in splenic T cells from *R26-LSL-CEPIAexternal*;*CD4-Cre* mice (red line) and *R26-LSL-CEPIAexternal*;*Itgal-LSL-Clover*;*Itgb2-LSL-mRuby2*;*CD4-Cre* (green line). The solid lines represent the mean; shaded areas, s.e.m. n = 30 cells from 3 experiments.

Source data are provided as a Source Data file.

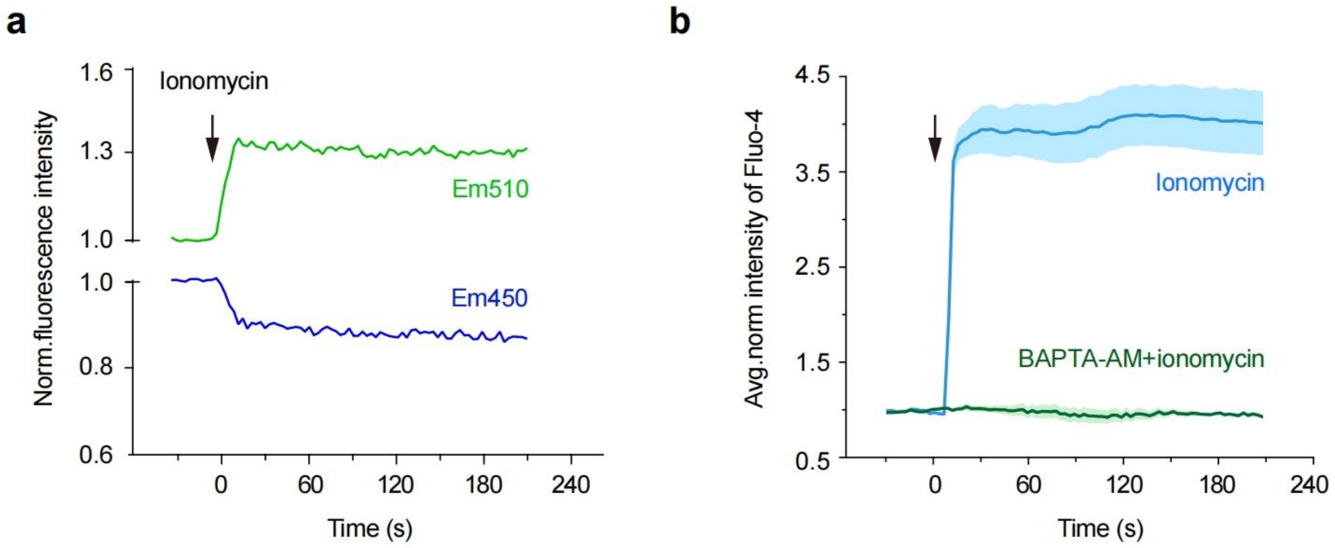

Supplementary Fig. 3: Effects of ionomycin treatment on CEPIA*external* and cytosolic Ca^{2+} dynamics in T cells.

Splenic T cells were isolated from *R26-LSL-CEPIAexternal*;*Itgal-LSL-Clover*;*Itgb2-LSL-mRuby2*;*CD4-Cre* mice and suspended in buffer containing 1.2 mM Ca^{2+} and 0.6 mM Mg^{2+}. Ionomycin (final concentration 1 μM) were added at time point 0.

a, Representative traces of CEPIA*external* dynamics in Em450 and Em510 channels of cell fluorescence intensity in response to stimulation with 1 μM ionomycin, which were normalized to the mean value of cells before addition of stimuli (related to Fig. 3b).

b, Cytosolic Ca^{2+} ($[Ca^{2+}]_{cyto}$) change was detected by Fluo-4 in mouse splenic T cells pretreated with 100 μM BAPTA-AM or DMSO vehicle control in response to stimulation with 1 μM ionomycin. The solid lines represent the mean; shaded areas, s.e.m. *n* = 30 cells from 3 experiments.

Source data are provided as a Source Data file.

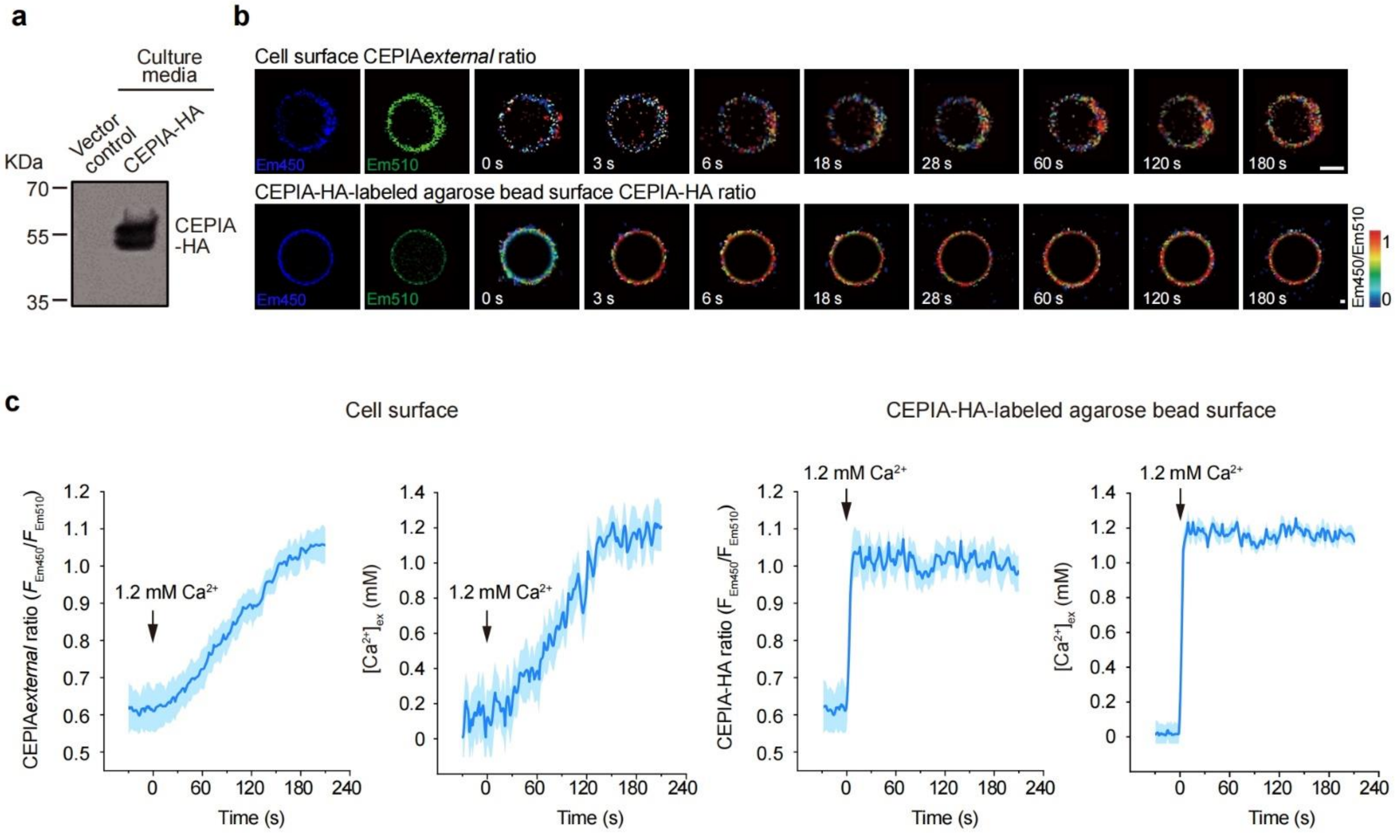

Supplementary Fig. 4: Ca^{2+} in the solution diffuses more quickly to the surface of agarose beads than to cell surface.

a, Immunoblot analysis of soluble CEPIA-HA protein with anti-HA tag antibody in culture media of 293T cells transiently transfected with CEPIA-HA or vector control. Images are from one representative experiment out of three.

b-c, Splenic T cells isolated from *R26-LSL-CEPIAexternal*;*Itgal-LSL-Clover*;*Itgb2-LSL-mRuby2*;*CD4-Cre* mice or CEPIA-HA-labeled anti-HA agarose beads were suspended in buffer containing 0 mM Ca^{2+} and 0.6 mM Mg^{2+}. 1.2 mM Ca^{2+} (final concentration) were added at time point 0.

b, Representative pseudocolour images of CEPIA*external* ratio on the surface of T cells or CEPIA-HA ratio on the surface of CEPIA-HA-labeled agarose beads in response to addition of Ca^{2+}. The first two images show the distribution of CEPIA*external* and CEPIA-HA in Em450 and Em510 channels. Scale bar, 3 μm. Images are from one representative experiment out of three.

c, Time course of CEPIA*external* ratio change and the corresponding change of $[Ca^{2+}]_{ex}$ on T cell surface or changes of CEPIA-HA ratio and $[Ca^{2+}]_{ex}$ on CEPIA-HA-labeled agarose bead surface in response to addition of Ca^{2+}.

The solid lines represent the mean; shaded areas, s.e.m. in **c**. n = 12 cells and n = 12 beads from 3 experiments. Source data are provided as a Source Data file.

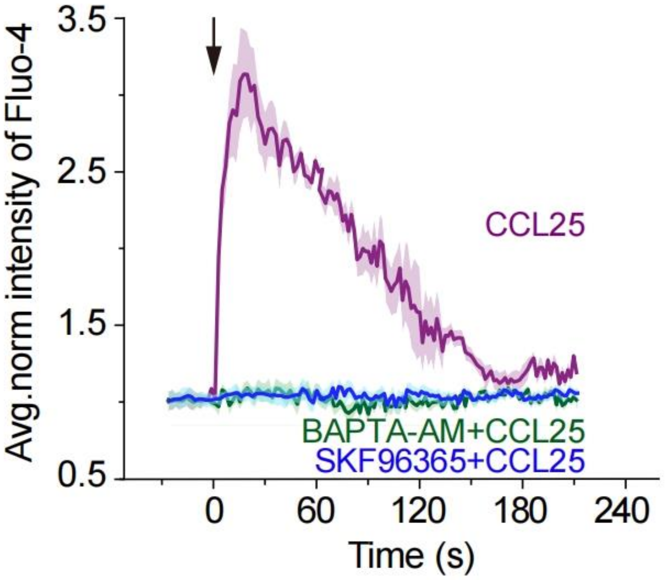

Supplementary Fig. 5: Cytosolic Ca^{2+} ($[Ca^{2+}]_{cyto}$) change in mouse splenic T cells pretreated with 100 µM BAPTA-AM, 100 µM SKF96365 or vehicle control in response to stimulation with 0.5 µg/ml CCL25 was detected by Fluo-4.

Splenic T cells were isolated from *R26-LSL-CEPIAexternal*;*Itgal-LSL-Clover*;*Itgb2-LSL-mRuby2*;*CD4-Cre* mice and suspended in buffer containing 1.2 mM Ca^{2+} and 0.6 mM Mg^{2+}. CCL25 (final concentration 0.5 µg/ml) were added at time point 0.

The solid lines represent the mean; shaded areas, s.e.m. n = 30 cells from 3 experiments. Source data are provided as a Source Data file.

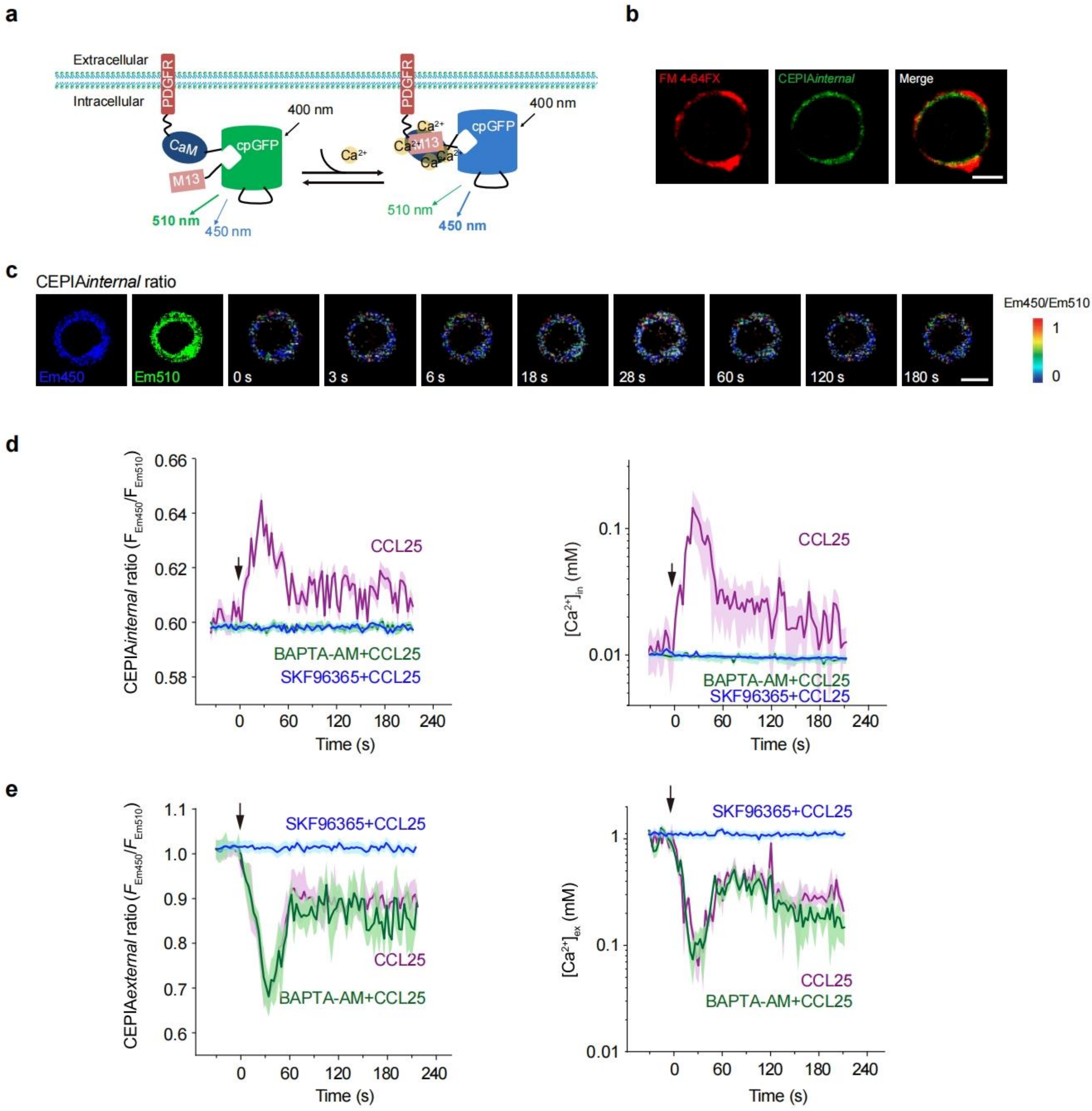

Supplementary Fig. 6: Establishing a cell membrane-anchored CEPIA*internal* to monitor intracellular submembrane Ca^{2+} ($[Ca^{2+}]_{in}$) dynamics.

a, Schematic diagram of the experimental setup for the membrane-anchored CEPIA*internal*.

b-d, Splenic T cells from WT mice transfected with CEPIA*internal* were suspended in buffer containing 1.2 mM Ca^{2+} and 0.6 mM Mg^{2+}. CCL25 (final concentration 0.5 µg/ml) were added at time point 0.

b, Representative fluorescence images of T cells showing the distribution of CEPIA*internal*. The plasma membrane as indicated by FM 4-64FX. Scale bar, 3 µm. Images are from one representative experiment out of three.

c, Representative pseudocolour images of CEPIA*internal* ratio in response to stimulation with 0.5 µg/ml CCL25. The first two images show the distribution of CEPIA*internal* in Em450 and Em510 channels. Scale bar, 3 µm. Images are from one representative experiment out of three.

d, Time course of CEPIA*internal* ratio change and the corresponding change of $[Ca^{2+}]_{in}$ in T cells pretreated with 100 µM BAPTA-AM, 100 µM SKF96365 or vehicle control in response to stimulation with 0.5 µg/ml CCL25.

e, Splenic T cells isolated from *R26-LSL-CEPIAexternal*;*Itgal-LSL-Clover*;*Itgb2-LSL-mRuby2*;*CD4-Cre* mice were suspended in buffer containing 1.2 mM Ca^{2+} and 0.6 mM Mg^{2+}. CCL25 (final concentration 0.5 µg/ml) were added at time point 0. Time course of CEPIA*external* ratio change and the corresponding change of $[Ca^{2+}]_{ex}$ in T cells pretreated with 100 µM BAPTA-AM, 100 µM SKF96365 or vehicle control in response to stimulation with 0.5 µg/ml CCL25.

The solid lines represent the mean; shaded areas, s.e.m. in **d** and **e**. *n* = 12 cells from 3 experiments. Source data are provided as a Source Data file.

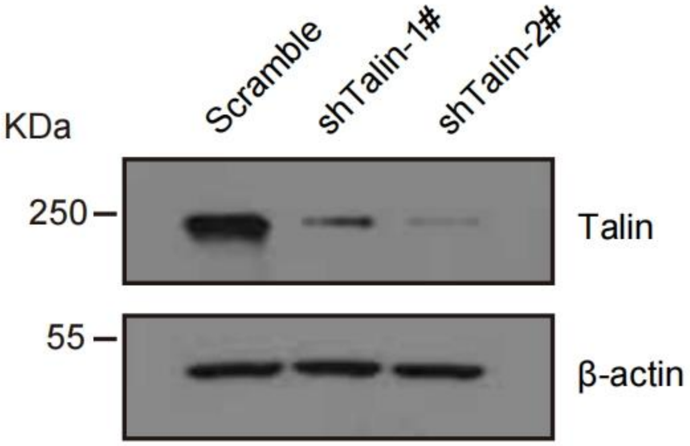

Supplementary Fig. 7: Talin knockdown in mouse splenic T cells.

Western blot analysis of talin silencing with shRNA in mouse splenic T cells isolated from *R26-LSL-CEPIAexternal*;*Itgal-LSL-Clover*;*Itgb2-LSL-mRuby2*;*CD4-Cre* mice. shTalin-2# was selected to generate T cells with talin knockdown in Fig. 7. Images are from one representative experiment out of three. Source data are provided as a Source Data file.

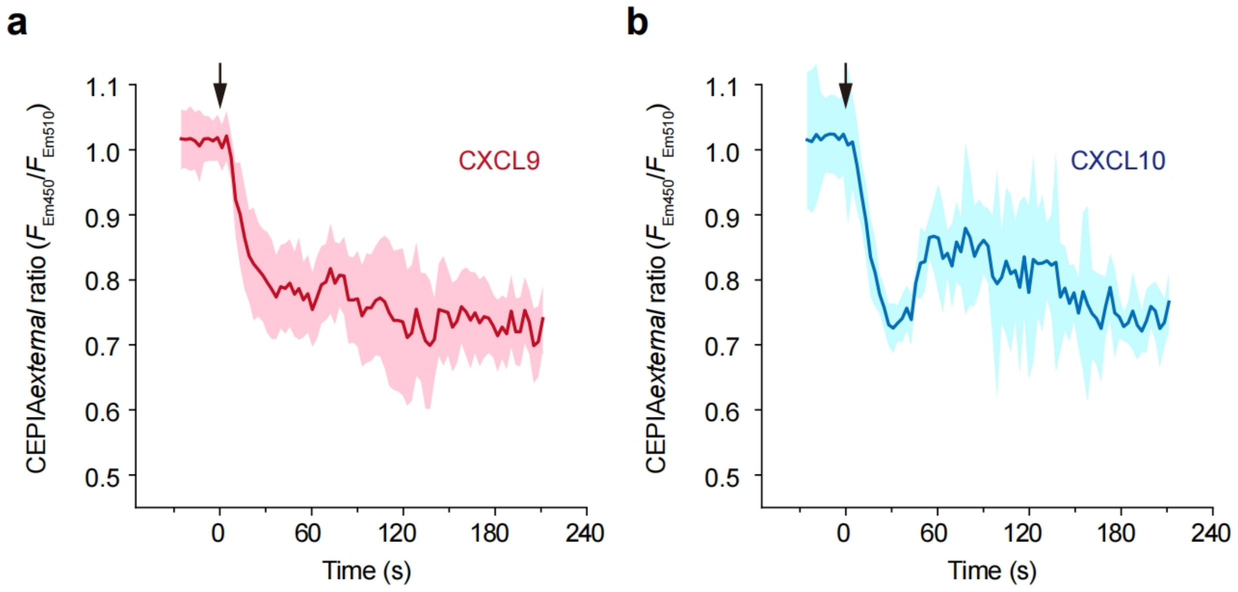

Supplementary Fig. 8: [Ca^{2+}]$_{ex}$ dynamics induced by CXCL9 and CXCL10.

Time course of CEPIA*external* ratio change in T cells in response to stimulation with 0.5 µg/ml CXCL9 (**a**) or CXCL10 (**b**).

The solid lines represent the mean; shaded areas, s.e.m. *n* = 30 cells from 3 experiments. Source data are provided as a Source Data file.
