# Supplementary material for: Ca2+ transients on the T cell surface trigger rapid integrin activation in a timescale of seconds
Source: Nat Commun. 2024 Jul 20;15:6131. doi: 10.1038/s41467-024-50464-0 (PMC11271479; doi:10.1038/s41467-024-50464-0)
Supplement: Supplementary file 3 — Description of Additional Supplementary Files [file 41467_2024_50464_MOESM3_ESM.pdf]

Description of Additional Supplementary Files

Supplementary Movie 1. Two-photon intravital imaging of the CEPIA*external* expressing T cells in the skin venules (up, raw image; down, CEPIA*external* fluorescence changes during T cell rolling), related to Fig. 8a,b.

Cell-tracer 647 signal (red), CEPIA*external* Em510 signal (green) and CEPIA*external* Em450 signal (blue) triple positive cells are shown. Vessels are delimited by iso-surface pattern obtained based on dextran and $CD31^+$ signals (red). Fluorescence signal changes are reflected by colour changes. Red signal diminishes due to the fluorescence quenching when T cells adherent to vessels. Colour bar represents fluorescence intensity, warmer colours indicate higher intensity. Images were collected at a rate of 1 FPS with 512×512 pixels. Data are representative of three independent experiments. Time shown in min:s.

Supplementary Movie 2. Example of slow rolling cells in the skin venules, related to Fig. 8c. CEPIA*external* Em450 signal (up, blue) and CEPIA*external* Em510 signal (down, green) positive T cells are shown. Em510 signal in a slow rolling T cell starts to show up in the beginning of the movie and gradually increases until T cell arrest. Images were collected at a rate of 20 FPS with 256×256 pixels, and the video plays at 0.25× speed. Data are representative of three independent experiments. Time shown in min:s.

Supplementary Movie 3. Two-photon intravital imaging of Cell-tracer 647 labelled control (left) or SKF96365-pretreated T cells in the skin venules, related to Fig. 9b.

T cells were pretreated with or without SKF96365 for 30 min and injected to recipient mice. Vessels are identified by dextran Texas Red and CD31-Alexa Fluor 594 (red). T cells are labelled with Cell-tracer 647 (red). The region within the rectangle highlights the ultimately arrested cells, each spot represents a single cell. Data are representative of three independent experiments. Time shown in min:s.
