# Supplementary material for: Ca2+ transients on the T cell surface trigger rapid integrin activation in a timescale of seconds
Source: Nat Commun. 2024 Jul 20;15:6131. doi: 10.1038/s41467-024-50464-0 (PMC11271479; doi:10.1038/s41467-024-50464-0)
Supplement: Supplementary file 7 — Reporting Summary [file 41467_2024_50464_MOESM7_ESM.pdf]

nature portfolio

Corresponding author(s): JianFeng Chen
ChangDong Lin

Last updated by author(s): Jun 16, 2024

Reporting Summary

Nature Portfolio wishes to improve the reproducibility of the work that we publish. This form provides structure for consistency and transparency in reporting. For further information on Nature Portfolio policies, see our Editorial Policies and the Editorial Policy Checklist.

Statistics

For all statistical analyses, confirm that the following items are present in the figure legend, table legend, main text, or Methods section.

n/a	Confirmed	
☐	☒	The exact sample size (*n*) for each experimental group/condition, given as a discrete number and unit of measurement
☐	☒	A statement on whether measurements were taken from distinct samples or whether the same sample was measured repeatedly
☐	☒	The statistical test(s) used AND whether they are one- or two-sided<br>*Only common tests should be described solely by name; describe more complex techniques in the Methods section.*
☒	☐	A description of all covariates tested
☐	☒	A description of any assumptions or corrections, such as tests of normality and adjustment for multiple comparisons
☐	☒	A full description of the statistical parameters including central tendency (e.g. means) or other basic estimates (e.g. regression coefficient) AND variation (e.g. standard deviation) or associated estimates of uncertainty (e.g. confidence intervals)
☐	☒	For null hypothesis testing, the test statistic (e.g. *F*, *t*, *r*) with confidence intervals, effect sizes, degrees of freedom and *P* value noted<br>*Give P values as exact values whenever suitable.*
☒	☐	For Bayesian analysis, information on the choice of priors and Markov chain Monte Carlo settings
☒	☐	For hierarchical and complex designs, identification of the appropriate level for tests and full reporting of outcomes
☒	☐	Estimates of effect sizes (e.g. Cohen's *d*, Pearson's *r*), indicating how they were calculated

Our web collection on statistics for biologists contains articles on many of the points above.

Software and code

Policy information about availability of computer code

Data collection	Nikon A1 confocal was used for fluorescent data collection.
Data analysis	Raw imaging data were analyzed using NIS-Elements software (Nikon, version 5.02.01) or ImageJ v1.8.0 as described in the Methods. Data from other experiments were analyzed by Origin 8.0. In vivo imaging data were analyzed by Imaris v9.5. Statistical analyses were performed using GraphPad Prism 9.

For manuscripts utilizing custom algorithms or software that are central to the research but not yet described in published literature, software must be made available to editors and reviewers. We strongly encourage code deposition in a community repository (e.g. GitHub). See the Nature Portfolio guidelines for submitting code & software for further information.

Data

Policy information about availability of data

All manuscripts must include a data availability statement. This statement should provide the following information, where applicable:

- Accession codes, unique identifiers, or web links for publicly available datasets
- A description of any restrictions on data availability
- For clinical datasets or third party data, please ensure that the statement adheres to our policy

All data generated in this study are available in the paper in Source Data file. Source data are provided with this paper.

Research involving human participants, their data, or biological material

Policy information about studies with human participants or human data. See also policy information about sex, gender (identity/presentation), and sexual orientation and race, ethnicity and racism.

Reporting on sex and gender	NA
Reporting on race, ethnicity, or other socially relevant groupings	NA
Population characteristics	NA
Recruitment	NA
Ethics oversight	NA

Note that full information on the approval of the study protocol must also be provided in the manuscript.

Field-specific reporting

Please select the one below that is the best fit for your research. If you are not sure, read the appropriate sections before making your selection.

[x] Life sciences [ ] Behavioural & social sciences [ ] Ecological, evolutionary & environmental sciences

For a reference copy of the document with all sections, see nature.com/documents/nr-reporting-summary-flat.pdf

Life sciences study design

All studies must disclose on these points even when the disclosure is negative.

Sample size	For all experiments, n≥3 was used according to standard scientific conventions, each sample size was described in detail in each figure legend. No statistical methods were used to predetermine the sample size.
Data exclusions	Cells that did not respond to stimulation were excluded from analysis. All exclusion criteria were pre-determined.
Replication	Unless otherwise noted, all experiments were repeated at least three times. All replication attempts were successful.
Randomization	Male and female mice at the defined age were randomly assigned to different experiment groups. Cells were randomly seeded into experimental groups after resuspension.
Blinding	All the experiments were performed without prior knowledge of the final outcome, and therefore blinding was not applied.

Reporting for specific materials, systems and methods

We require information from authors about some types of materials, experimental systems and methods used in many studies. Here, indicate whether each material, system or method listed is relevant to your study. If you are not sure if a list item applies to your research, read the appropriate section before selecting a response.

Materials & experimental systems

n/a	Involved in the study	
[ ]	[x]	Antibodies
[ ]	[x]	Eukaryotic cell lines
[x]	[ ]	Palaeontology and archaeology
[ ]	[x]	Animals and other organisms
[x]	[ ]	Clinical data
[x]	[ ]	Dual use research of concern
[x]	[ ]	Plants

Methods

n/a	Involved in the study	
[x]	[ ]	ChIP-seq
[x]	[ ]	Flow cytometry
[x]	[ ]	MRI-based neuroimaging

Antibodies

Antibodies used	Primary antibodies used were:<br>Rabbit anti-integrin β1 (1:2000, Abcam, Catalog No.: Ab52971, Lot No.: GR162323-9),<br>Rabbit anti-HA tag (1:1000, Cell Signaling Technology, Catalog No.: 3724S, Lot No.: 8),<br>Mouse anti-β-actin (1:5000, ABclonal, Catalog No.: AC004, Lot No.: 3523031406),

	Mouse anti-Talin (1:500, Sigma-Aldrich, Catalog No.: T3287, Lot No.: 049M4782V).<br>Secondary antibodies used were:<br>Goat Anti-Mouse IgG (H+L) HRP (1:5000, Multi Sciences, Catalog No.: GAM0072, Lot No.: A30821),<br>Goat Anti-Rabbit IgG (H+L) HRP (1:5000, Multi Sciences, Catalog No.: GAR0072, Lot No.: A30351).
Validation	β1 (Abcam Ab52971), documented to papers with PMID: 33982786, 34108040, 32157808<br>HA tag (Cell Signaling Technology 3724S), documented to papers with PMID: 38228802, 38238430, 38277122<br>β-actin (ABclonal AC004), documented to papers with PMID: 32238924, 33991488, 30729032<br>Talin (Sigma-Aldrich T3287), documented to papers with PMID: 23620790, 26336988, 19093722<br>Goat Anti-Mouse IgG (H+L) HRP (Multi Sciences GAM0072), documented to papers with PMID: 34886671, 34623756, 34977528<br>Goat Anti-Rabbit IgG (H+L) HRP (Multi Sciences GAR0072), documented to papers with PMID: 27943419, 28266177, 26788137

Eukaryotic cell lines

Policy information about cell lines and Sex and Gender in Research

Cell line source(s)	293T cells used in this study are purchased from ATCC (Manassas, VA).
Authentication	None of the cell lines were authenticated since they were commercial samples supplied by ATCC.
Mycoplasma contamination	Cell lines were tested for mycoplasma contamination and were found negative.
Commonly misidentified lines (See ICLAC register)	No commonly misidentified cell lines were used in this study.

Animals and other research organisms

Policy information about studies involving animals; ARRIVE guidelines recommended for reporting animal research, and Sex and Gender in Research

Laboratory animals	All mice were of the C57BL/6J background. WT C57BL/6J mice and CD4-Cre mice were obtained from Jackson Laboratory. Itgal-loxP-Stop-loxP-Clover, Itgb2-loxP-Stop-loxP-mRuby2 and Rosa26-loxP-Stop-loxP-CEPIAexternal C57BL/6J mice were generated by Shanghai Biomodel Organism Science & Technology Development Co., Ltd. 7-12 week-old mice (both male and female) were used in this study. All mice were kept in group housing (2-5 mice per cage) in a specific pathogen-free facility with controlled environmental conditions of temperature (20-25℃), humidity (30-70%) and light (a 12-h light/dark cycle) at Center for Excellence in Molecular Cell Science, Chinese Academy of Sciences.
Wild animals	The study did not involve wild animals.
Reporting on sex	The study had no sex selectivity in the mice. Sex and age-matched mice were analyzed and reported as aggregate as no phenotypic link to sex was detected.
Field-collected samples	The study did not involve samples collected from the field.
Ethics oversight	All experiments were conducted under protocols (SIBCB-S323-1802-005) approved by the Institutional Animal Care and Use Committees (IACUCs) of Center for Excellence in Molecular Cell Science, Chinese Academy of Sciences. Euthanasia was performed using CO2 asphyxiation.

Note that full information on the approval of the study protocol must also be provided in the manuscript.

Plants

Seed stocks	NA
Novel plant genotypes	NA
Authentication	NA
